# Male-specific lethal 1 (MSL1) promotes Erastin-induced ferroptosis in colon cancer cells by regulating the KCTD12-SLC7A11 axis

**DOI:** 10.1038/s41419-025-07555-7

**Published:** 2025-04-12

**Authors:** Lifu Luo, Qingzhi Zhao, Xueli Cui, Shijiao Dong, Yong Wang, Nan Jiang, Chengyu Cai, Jingji Jin, Bing Liang

**Affiliations:** 1 Department of Ophthalmology, The Second Hospital of Jilin University, Changchun, Jilin Province China; 2 School of Nursing, Jilin University, Changchun, Jilin Province China; 3 School of Life Sciences, Jilin University, Changchun, Jilin Province China; 4 Urology Department, Jilin Province People’s Hospital, Changchun, Jilin Province China; 5 Department of Cardiovascular center, Jilin University First Hospital, Changchun, Jilin Province China

**Keywords:** Colon cancer, Preclinical research

## Abstract

MSL1, a scaffold protein of the MSL histone acetyltransferase complex, is crucial for its structural integrity and enzymatic activity. While MSL1 is highly expressed in various tumors, its role in tumor progression and cell death remains unclear. Here, we provide evidence of a negative regulatory relationship between MSL1 and KCTD12 through biochemical assays and knockdown/overexpression studies. Notably, in colon cancer cells, the ferroptosis inducer Erastin significantly suppressed MSL1 expression, leading to KCTD12 upregulation. Moreover, MSL1 promotes Erastin-induced ferroptosis in HCT116 and SW480 cells via the KCTD12-SLC7A11 axis. Consistently, Erastin-induced changes in ROS, GSH, and MDA levels were regulated by this axis, highlighting its role in ferroptosis. These findings offer potential therapeutic targets and a theoretical foundation for colon cancer treatment.

## Introduction

Gene dosage compensation ensures balanced X-chromosome genes expression between males and females. In *Drosophila*, this process is mediated by the male-specific lethal (MSL) complex, composed of five proteins and two long non-coding RNAs [1]. In contrast, mammals retain only four highly conserved subunits: MOF, MSL1, MSL2, and MSL3 [2]. Among them, MSL1 acts as a scaffold protein, assembling the MSL complex and facilitating histone H4 acetylation at lysine 16 (H4K16ac) [3]. Beyond its role in dosage compensation [4], MSL1 also regulates cell cycle progression and proliferation [5]. Recently, its regulatory role in tumor cells has gained attention [6, 7]. While studies suggest that MSL1 inhibition enhances chemotherapy-induced cytotoxicity in cancer cells [6], the underlying molecular mechanisms remain unclear.

Our preliminary data indicate a potential link between MSL1 and KCTD12, which has not been previously reported. KCTD12 (Pfetin), a member of the KCTD family, is involved in GABA_B2_ receptor signaling [8], Wnt-Notch pathway inhibition [9], and G2/M cell cycle transition [10]. Depending on tumor types, KCTD12 exhibits oncogenic or tumor-suppressive properties and serves as a prognostic biomarker in gastrointestinal tumors [11]. In colorectal cancer, its downregulation suggests a tumor-suppressive role [12], but the exact molecular mechanism remains unclear. Elucidating KCTD12’s role in colon cancer could provide new insights for targeted therapies and improve patient outcomes.

Effective cancer treatment aims to maximize tumor cell death [13]. Mesenchymal and dedifferentiated tumor cells often resist apoptosis but remain highly susceptible to ferroptosis [14–16], a regulated cell death pathway increasingly recognized in colon cancer [17, 18]. Several anticancer agents, including Apatinib [19], and honokiol [20], and *Camellia nitidissima Chi* extract [21], induce ferroptosis to inhibit tumor progression. The key ferroptosis regulator, SLC7A11, is overexpressed in colon cancer cells and tissues [22]. As part of the Xc- system, SLC7A11 forms a complex with SLC3A2, facilitating cystine uptake for glutathione (GSH) biosynthesis and protecting cells from oxidative stress and ferroptosis [23]. Thus, targeting SLC7A11 is a promising strategy for colon cancer therapy.

Although no studies directly link MSL1 or KCTD12 and ferroptosis, KCTD12 interacts with the metabolic glutamate receptor 1 (mGlu1) [24], which regulates glutamate (Glu) levels [25]. Since Glu serves as an equimolar exchanger for cystine in the Xc- system, KCTD12 may influence SLC7A11-mediated ferroptosis. Based on this, we hypothesize that MSL1 induces ferroptosis via SLC7A11 suppression, potentially inhibiting colon cancer progression through its interacting with KCTD12.

## Materials and methods

### Tissue collection

Pathological tissue samples from 40 colon cancer patients who underwent radical surgery at the Third Hospital of Jilin University (2017–2018) were collected without prior neoadjuvant radiotherapy or chemotherapy. Tumor and adjacent tissues (<2 cm away from the tumor site) were frozen for qPCR analysis. The patients had a median age of 59.8 years (range: 30–85).

### Cell culture and reagents

HEK239T, HCT116, and SW480 cell lines were obtained from the laboratory cell bank and cultured in DMEM or 1640 medium (MeilunBio, Dalian, China) with 10% fetal bovine serum. Cells were maintained at 37 °C in a humidified incubator with 5% CO_2_. All cell lines were authenticated by short tandem repeat profiling within the past three years and confirmed to be mycoplasma-free. Fer-1 was purchased from Selleckchem (Shanghai, China), and Erastin from AbMole (Shanghai, China).

### Transient transfection and establishment of stable cell lines

HEK293T, HCT116, and SW480 cells at 30–40% confluence in 6-well plates were transiently transfected with Flag-MSL1, Flag-KCTD12, pLVX-shMSL1 or pLVX-shKCTD12 plasmids using polyethyleneimine (PEI) (23966, Polysciences). Stable Flag-KCTD12-expressing HCT116 and OCM-1 cell lines were established as previously described [26] and confirmed by Western blot with an anti-Flag antibody.

### Lenti-viral mediated interference of gene expression

The pLVX-Puro-shRNA system was used to express shRNA in HEK293T, HCT116, and SW480 cells. The target sequence for MSL1 was: shMSL1, GATCCGCACCGGACGTGTAGGAAATTTCAAGAGAATTTCCTACACGTCCGGTGTTTTTTG. The pLVX-ZsGreen-shKCTD12 plasmid was synthesized by Sangon Biotech (Beijing, China). Knockdown efficiency was verified by western blot using anti-MSL1 and anti-KCTD12 antibodies after transient transfection of pLVX-shRNAs.

### Generation of the MSL1-KO and MSL3-KO cell lines

Generation of human MSL1 and MSL3 knockout cell lines was performed as previously described [27]. Knockout efficiency was confirmed by western blot, and indel mutations were verified by DNA sequencing.

### Western blot and antibodies

Proteins from whole-cell lysates were analyzed by western blot with specific antibodies and detected using a chemiluminescent system (Clinx Science Instruments). Band intensities were quantified using ImageJ software. Primary antibodies included MSL1 (24373-1-AP, Proteintech), Flag (F3165, Sigma-Aldrich), GPX4 (ab125066, Abcam), SLC7A11 (ab307601, Abcam), KCTD12 (15523-1-AP, Proteintech), H4K16ac (PTM-122, Jingjie Biotechnology), and GAPDH (raised against bacterially expressed protein, Jilin University).

### Reverse transcriptase PCR

The process was conducted as previously reported [28].

### ROS, GSH, and MDA assays

The ROS (S0033S), GSH (S0053), and MDA (S0131S) assay kits were from Beyotime (Shanghai, China), and the levels of ROS, GSH, and MDA were detected in transfected with different plasmids cells according to the manufacturer’s instructions.

### MTT assay

The process was carried out as previously reported by our group [29].

### Statistical analysis

All data were analyzed using SPSS 16.0 software (IBM, Armonk, New York, NY, USA). Data from at least three independent replicates were presented as the mean ± SD. Two groups were assessed by a *t*-test, and three or more groups were assessed by one-way analysis of variance (ANOVA). *p* < 0.05 was considered statistically significant.

## Results

### KCTD12 may be a downstream regulatory target of MSL1/MSL3

**Fig. 1:**
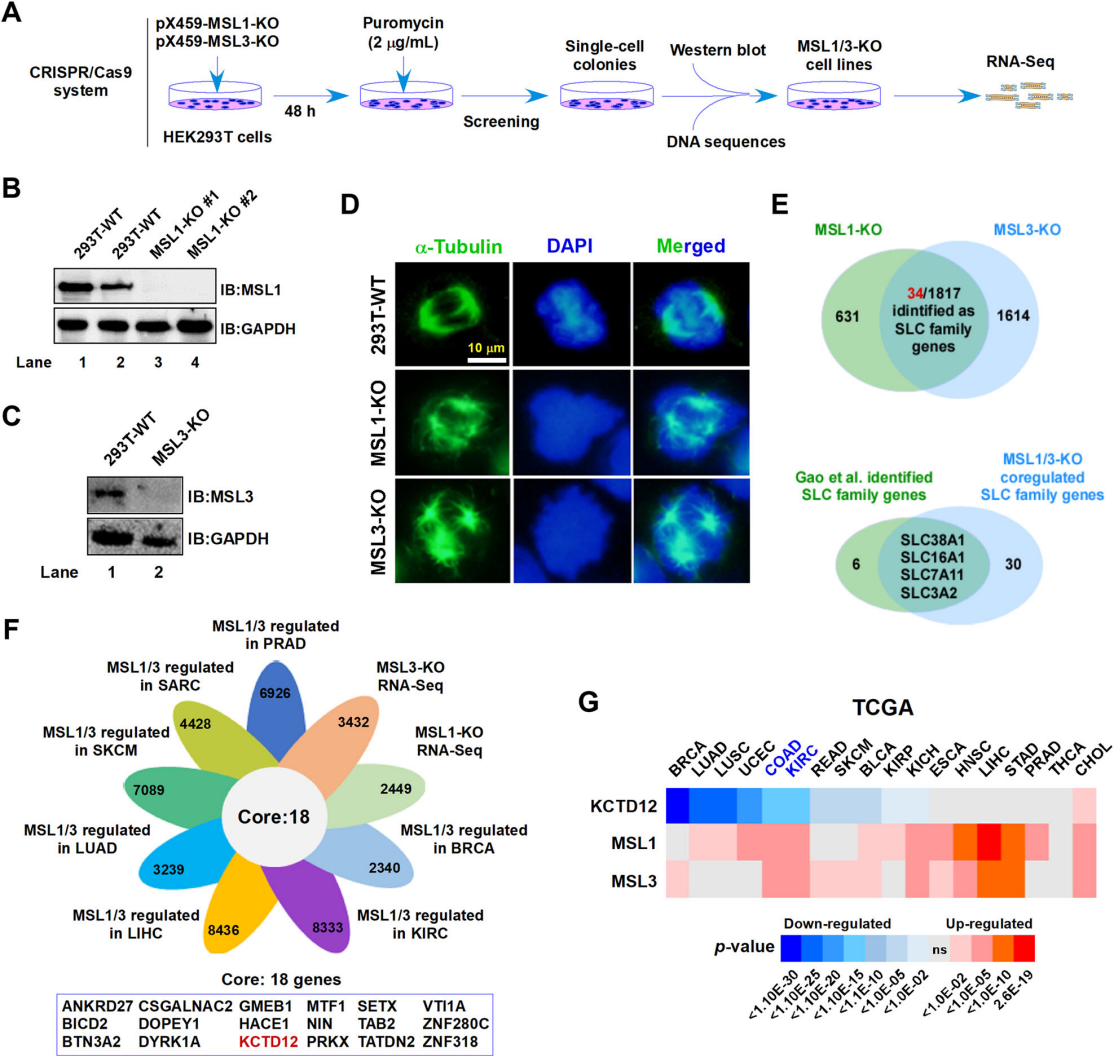
Potential correlation between MSL1/MSL3 and KCTD12. **A** Schematic representation of the procedural steps for establishing MSL1-KO and MSL3-KO 293T cell lines. **B**, **C** Western blot analysis confirmed the knockout efficiency of MSL1 and MSL3 using specific antibodies. GAPDH was used as an internal control. **D** Immunofluorescence staining of cells with α-Tubulin to assess the effects of MSL1-KO and MSL3-KO on cell division. **E** Venn diagram analysis of RNA-Seq data from MSL1-KO and MSL3-KO cells (upper panel), and overlapping analysis between ferroptosis-related SLC family genes and MSL1-KO/MSL3-KO coregulated SLC family genes (lower panel). **F** Identification of intersecting genes coregulated by MSL1/MSL3 across multiple tumor tissues. **G** Correlation analysis of KCTD12 with MSL1/MSL3 based on TCGA database.

We generated MSL1 and MSL3 knockout (KO) 293T cell lines using CRISPR/Cas9 (Fig. 1A) and assessed their effects on cell division via immunofluorescence staining. Both MSL1-KO (Fig. 1B) and MSL3-KO (Fig. 1C) cells exhibited spindle multipolarity (green, Fig. 1D), suggesting MSL1/MSL3 involvement in mitotic regulation. RNA-Seq analysis identified 2448 and 3431 differentially expressed genes (DEGs) in MSL1-KO and MSL3-KO cells, respectively, with 1817 overlapping DEGs. Notably, 34 of these belonged to the solute carrier (SLC) family, implying MSL1/MSL3-mediated regulation of SLC genes (Fig. 1E, upper). This aligns with findings from Gao et al., who identified 10 SLC genes associated with ferroptosis [30]. Four of these overlapped with the 34 SLC genes coregulated by MSL1/MSL3, linking MSL1/MSL3 to ferroptosis regulation (Fig. 1E, lower).

Further Venn analysis of MSL1/MSL3 coregulated genes (TCGA data) across tumors and DEGs from RNA-Seq revealed 18 overlapping genes (Fig. 1F). Among these, KCTD12 stood out, as our previous work demonstrated its inhibitory effect on OCM-1 cell proliferation [26]. TCGA analysis showed that KCTD12 expression was generally lower in tumors, while MSL1/MSL3 were highly expressed, suggesting a potential inverse correlation in COAD and KIRC tissues (Table 1 and Fig. 1G).

**Table 1:** Differentially expressed MSL1, MSL3, and KCTD12 genes in various tumor tissues.

Tumors	KCTD12 (*P*-value)	Regulated type	MSL1 (*P*-value)	Regulated type	MSL3 (*P*-value)	Regulated type
BRCA	1.17E-51	Down	0.14378164	ns	0.000532	Up
LUAD	2.14E-27	Down	0.00247984	Up	0.823263	ns
LUSC	2.03E-29	Down	0.00394354	Up	0.317808	ns
UCEC.	8.06E-21	Down	1.29E-07	Up	0.096974	ns
COAD	1.45E-15	Down	7.30E-08	Up	3.88E-09	Up
KIRC	3.43E-16	Down	1.15E-05	Up	5.33E-09	Up
READ	8.76E-07	Down	0.15319792	ns	0.011912	Up
SKCM	2.86E-07	Down	0.10306048	ns	0.000118	Up
BLCA	1.81E-06	Down	0.04491664	Up	0.004616	Up
KIRP	0.02023342	Down	0.00027205	Up	0.344453	ns
KICH	0.05205218	Down	7.71E-09	Up	2.82E-08	Up
ESCA	0.63818056	ns	4.03E-05	Up	0.000269	Up
HNSC	0.66916146	ns	1.14E-12	Up	9.64E-07	Up
LIHC	0.46169307	ns	2.60E-19	Up	2.01E-11	Up
STAD	0.12251715	ns	5.89E-13	Up	2.12E-13	Up
PRAD	0.43480534	ns	1.18E-07	Up	0.109063	ns
THCA	0.70438965	ns	0.25788699	ns	0.14111	ns
CHOL	0.00222305	Down	1.58E-08	Up	2.26E-09	Up

*P*-value, represents the difference in gene expression compared to normal tissues.

*BRCA* breast cancer, *LUAD* lung adenocarcinoma, *LUSC* lung squamous cell carcinoma, *UCEC* endometrioid cancer, *COAD* colon cancer, *KIRC* kidney clear cell carcinoma, *READ* rectal cancer, *SKCM* skin melanoma, *BLCA* bladder cancer, *KIRP* kidney papillary cell carcinoma, *KICH* kidney chromophobe, *ESCA* esophageal cancer, *HNSC* head and neck cancer, *LIHC* liver cancer, *STAD* stomach cancer, *PRAD* prostate cancer, *THCA* thyroid cancer, *CHOL* cholangiocarcinoma.

### Downregulation of KCTD12 in primary colon cancer tissues

**Fig. 2:**
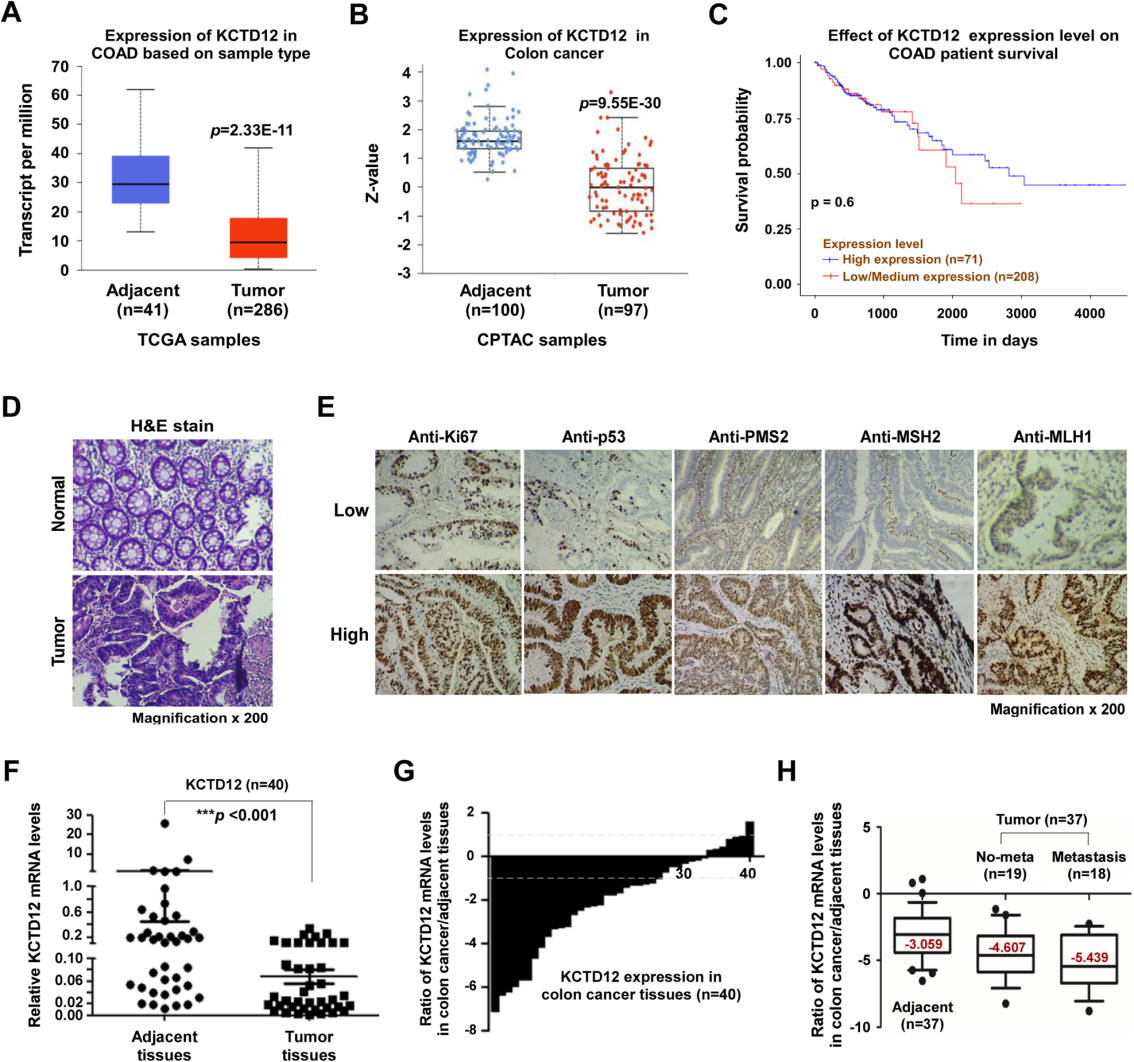
Frequent downregulation of KCTD12 in primary diagnosed colon cancer tissues. KCTD12 expression at the mRNA (**A**) and protein (**B**) levels in colon cancer tissues, based on TCGA database analysis. **C** Correlation between KCTD12 expression and patient survival. **D** H&E staining of colon cancer tissues (magnification, ×200). **E** IHC staining for Ki67, p53, PMS2, MSH2 and MLH1 in colon cancer tissues, showing low (upper) and high (lower) expression patterns. **F** KCTD12 expression in tumor and adjacent tissues from 40 colon cancer patients, normalized to GAPDH. Data are presented using mean ± standard deviation; ***p < 0.001 vs. adjacent tissues. **G**, **H** Expression patterns of KCTD12 in colon cancer. Each bar presents the log_2_ ratio of KCTD12 expression between tumors and matched adjacent tissues (*n* = 40). Bars >1 indicate a >2-fold increase, while bars value <1 indicate a >2-fold decrease. Each bar represents the means of three independent experiments. The red number in **H** denotes the median value.

TCGA data revealed a significant downregulation of KCTD12 at both mRNA (Fig. 2A) and protein (Fig. 2B) levels in colon cancer compared to normal tissues. Notably, low KCTD12 expression correlated with poorer patient survival (Fig. 2C). To validate these findings, paired tumor and adjacent tissues were collected from 40 patients (24 males and 16 females). H&E staining images are shown in Fig. 2D, while immunohistochemical staining of Ki67, p53, PMS2, MSH2, and MLH1—common diagnostic markers—is presented in Fig. 2E. KCTD12 mRNA levels were significantly lower in tumors than in matched adjacent tissues (Fig. 2F), with 67.5% (27/40) of patients exhibiting a > 2-fold decreased (Fig. 2G). Notably, KCTD12 downregulation was more pronounced in patients with distant metastasis (Fig. 2H).

### MSL1 negatively regulates KCTD12 in 293 T and colon cancer cells

**Fig. 3:**
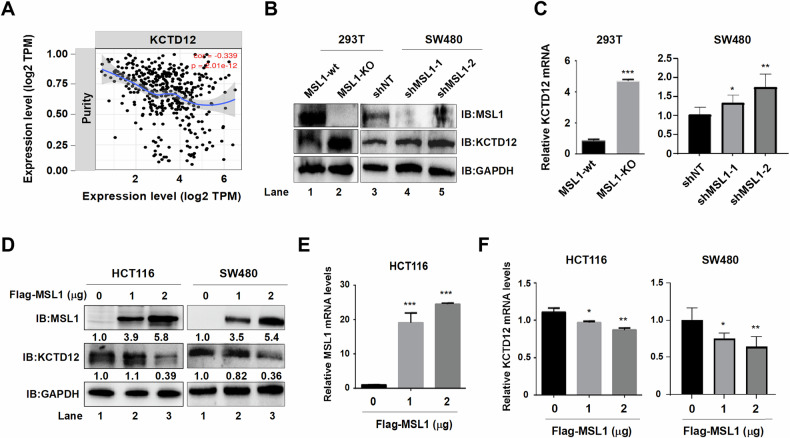
MSL1 negatively regulates KCTD12 in 293 T and colon cancer cells. **A** Correlation analysis between MSL1 and KCTD12 using TIMER platform (https://cistrome.shinyapps.io/timer/). **B**, **C** KCTD12 expression at the protein and mRNA levels in wild type and MSL1-KO 293T cells, as well as in shNT and shMSL1 SW480 cells. **D**–**F** Overexpression of Flag-MSL1 in HCT116 and SW480 cells reduced KCTD12 protein and mRNA levels in a dose-dependent manner. *p < 0.05, **p < 0.01, ***p < 0.001 vs. control.

Given MSL1’s role as a core MSL complex subunit, we focused on its interaction with KCTD12. TIMER analysis confirmed a negative correlation between MSL1 and KCTD12 in colon cancer cells (cor = −0.339, Fig. 3A). Consistently, KCTD12 expression was significantly upregulated at both protein (Fig. 3B) and mRNA (Fig. 3C) levels in MSL1-KO and shMSL1 cells. Conversely, MSL1 overexpression led to a marked reduction in KCTD12 protein and mRNA levels (Fig. 3D–F), demonstrating that MSL1 negatively regulates KCTD12.

### SLC7A11 as a potential target of the MSL1-KCTD12 axis

**Fig. 4:**
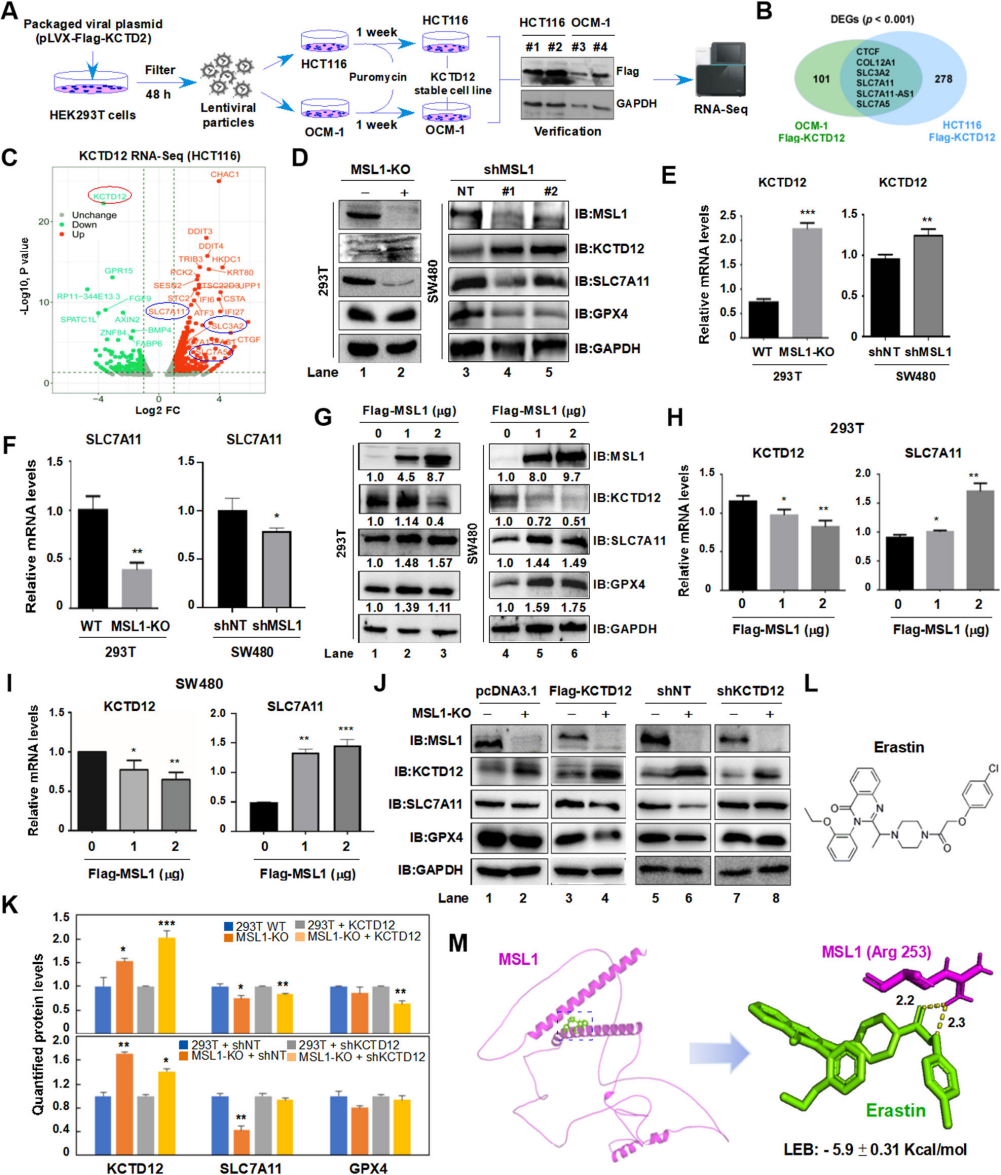
The MSL1-KCTD12 axis regulates SLC7A11 in 293 T and colon cancer cells. **A** Schematic workflow for generating Flag-KCTD12 stable cell lines. **B** Venn diagram of DEGs (*p* < 0.001) from RNA-Seq analysis in KCTD12-overexpressing HCT116 and OCM-1 cells. **C** Volcano plot of DEGs (*p* < 0.01) in KCTD12-expressing HCT116 cells. **D** Western blot analysis of MSL1, KCTD12, SLC7A11, and GPX4 in wild-type vs. MSL1-KO 293 T cells and shNT vs. shMSL1 SW480 cells. **E**, **F** RT-qPCR analysis of KCTD12 and SLC7A11 mRNA levels. *p < 0.05, **p < 0.01, ***p < 0.001 vs. wild-ty*p*e. **G** Immunoblot analysis of KCTD12, SLC7A11, and GPX4 in Flag-MSL1- overexpressing (0, 1 and 2 μg) 293T and SW480 cells. **H**, **I** RT-qPCR quantification of KCTD12 and SLC7A11 mRNA levels in 293 T and SW480 cells. *p < 0.05, **p < 0.01, ***p < 0.001 vs. 0 μg group. **J** Western blot analysis of indicated proteins in wild-type and MSL1-KO 293 T cells after transient transfection or knockdown of KCTD12. **K** Quantification of relative protein levels in (**J**). *p < 0.05, **p < 0.01, ***p < 0.001 vs. vector or shNT. GAPDH served as a loading control. **L** Chemical structure of Erastin. **M** Molecular docking analysis of Erastin with MSL1.

RNA-Seq was performed on lentiviral-mediated KCTD12-overexpressing HCT116 and OCM-1 cells (Fig. 4A), identifying 284 and 107 DEGs (*p* < 0.001), respectively. Venn analysis revealed six overlapping genes including SLC7A11 (Fig. 4B). DEGs from Flag-KCTD12 HCT116 cells (*p* < 0.01) were visualized in volcano plot (Fig. 4C). Notably, SLC7A11 and SLC3A2 form the Xc- system, a key negative regulator of ferroptosis.

To investigate the link between SLC7A11 and the MSL1-KCTD12 axis, we examined SLC7A11 expression in MSL1-KO and shMSL1 cells. Higher KCTD12 levels were associated with reduced SLC7A11 expression at both the protein and mRNA levels (Fig. 4D–F). Conversely, MSL1 overexpression led to a dose-dependent decrease in KCTD12 and an increase in SLC7A11 expression (Fig. 4G–I). Furthermore, in MSL1-KO 293T cells, KCTD12 overexpression suppressed SLC7A11 and GPX4 expression (Fig. 4J, Lane 4), whereas shKCTD12 reversed this effect, restoring SLC7A11 levels (Lane 8 vs. Lane 7). GPX4 was similarly modulated by the MSL1-KCTD12 axis. Protein quantifications are shown in Fig. 4K.

Finally, molecular docking analysis using AutoDock Vina 1.1.2 and Amber14 identified binding sites between MSL1 and Erastin, as shown in Fig. 4L, M. These findings suggest that the MSL1-KCTD12 axis regulates SLC7A11 and may influence ferroptosis.

### MSL1-KTD12 axis modulates Erastin-induced ferroptosis via SLC7A11 in colon cancer cells

**Fig. 5:**
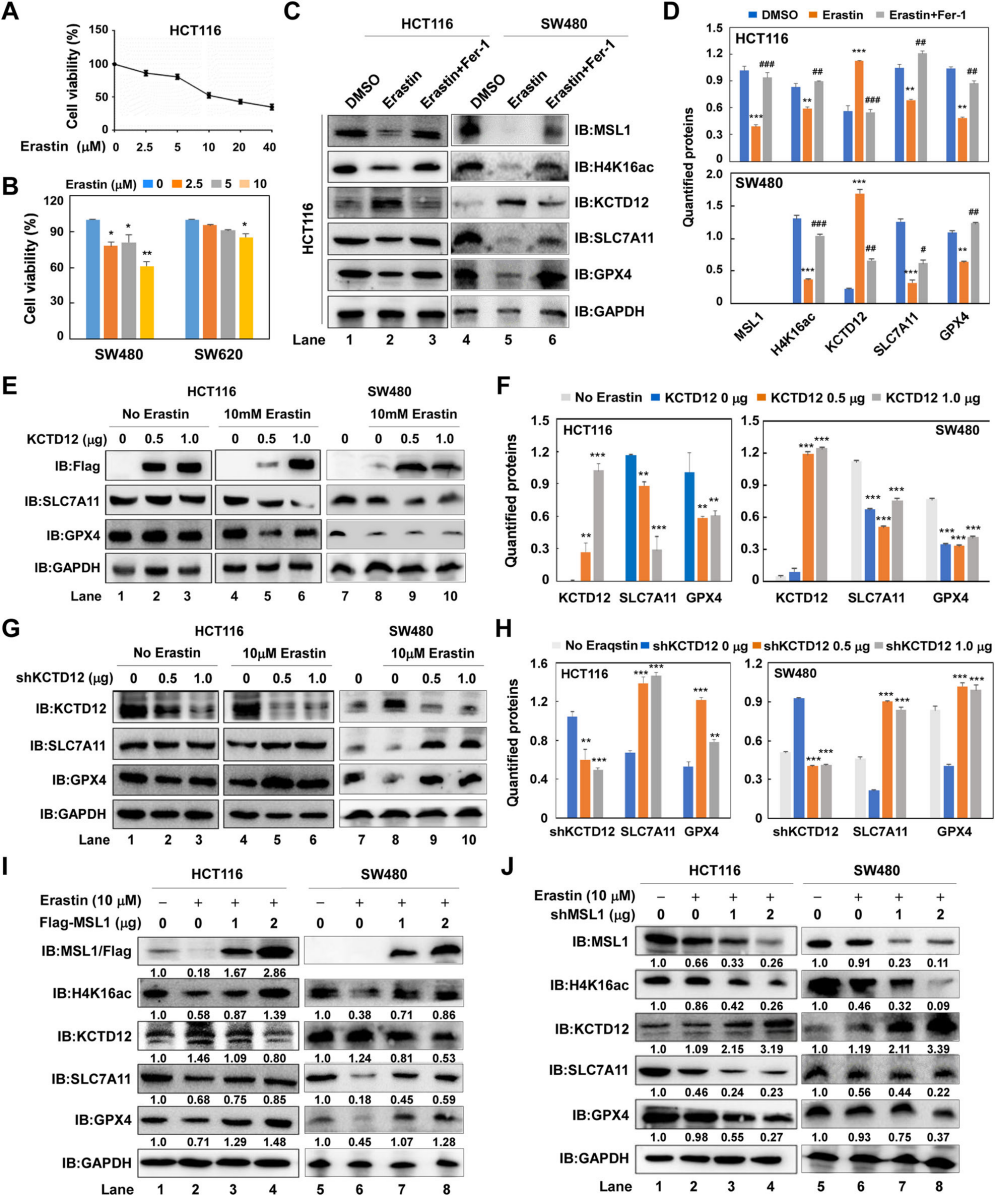
MSL1 regulates SLC7A11 via KCTD12 in Erastin-induced ferroptosis. **A** HCT116 cell viability was assessed by CCK-8 after 24 h Erastin treatment (0–40 µM). **B** Viability of SW480 and SW620 cells treated with Erastin (0–10 µM) for 24 h, measured by CCK-8 assay. **C** Western blot analysis of MSL1, KCTD12, H4K16ac, SLC7A11, and GPX4 in HCT116 and SW480 cells treated with 10 µM Erastin ± Fer-1. **D** Quantification of (**C**). **E** Immunoblot analysis of SLC7A11 and GPX4 in HCT116 and SW480 cells transiently transfected with Flag-KCTD12 (0–1.0 µg) in the presence or absence of 10 µM Erastin. **F** Quantification of (**E**). **G** Immunoblot analysis of SLC7A11 and GPX4 in HCT116 and SW480 cells transfected with pLVX-shKCTD12 (0–1.0 µg) ± 10 µM Erastin. **H** Quantification of (**G**). **I**, **J** Western blot analysis of indicated proteins in 10 µM Erastin-treated HCT116 and SW480 cells upon MSL1 overexpression or knockdown. Numbers below immunoblots represent quantified protein levels to the DMSO control. *p < 0.05, **p < 0.01, ***p < 0.001 vs. control.

Given that the MSL1-KTD12 axis regulates SLC7A11 expression, we hypothesized its involvement in ferroptosis. To test this, we first assessed the sensitivity of three different colon cancer cells to the class I ferroptosis inducer Erastin using MTT assays. Erastin significantly induced cell death in HCT116 and SW480 cells. (Fig. 5A, B). Immunoblot analysis revealed a marked reduction in MSL1 and H4K16ac levels in Erastin-treated cells, accompanied by increased KCTD12 and decreased SLA7A11 and GPX4 expression (Fig. 5C, Lane 2 and 5). These changes were reversed by Fer-1 treatment (Lane 3 and 6), as quantified in Fig. 5D.

Next, we examined the effect of KCTD12 on SLC7A11 expression in Erastin-treated and untreated cells. KCTD12 overexpression significantly reduced SLC7A11 and GPX4 protein level (Fig. 5E, F), whereas KCTD12 knockdown resulted in their upregulation during Erastin-induced ferroptosis (Fig. 5G, H), confirming its negative regulatory role. Similarly, in cells transiently transfected with Flag-MSL or pVLX-shMSL1, Erastin treatment led to a marked decrease in MSL1 and H4K16ac, consistent with previous findings (Fig. 5I, Lane 2 vs. Lane 1, Lane 6 vs. Lane 5). MSL1 depletion further enhanced KCTD12 expression while suppressing SLC7A11 and GPX4 (Fig. 5J, Lane 3–4 vs. Lane 2; Lane 7–8 vs. Lane 6). Conversely, MSL1 overexpression restored SLC7A11 and GPX4 levels in a dose-dependent manner (Fig. 5I, Lane 3–4 vs. Lane 1; Lane 7–8 vs. Lane 5). Quantified protein levels are indicated in the immunoblots. Collectively, these findings demonstrate that the MSL1-KCTD12 axis regulates SLC7A11 and GPX4, thereby modulating Erastin-induced ferroptosis in colon cancer cells.

### MSL1 and KTD12 differentially regulate Erastin-induced GSH, MDA, and ROS levels in colon cancer cells

**Fig. 6:**
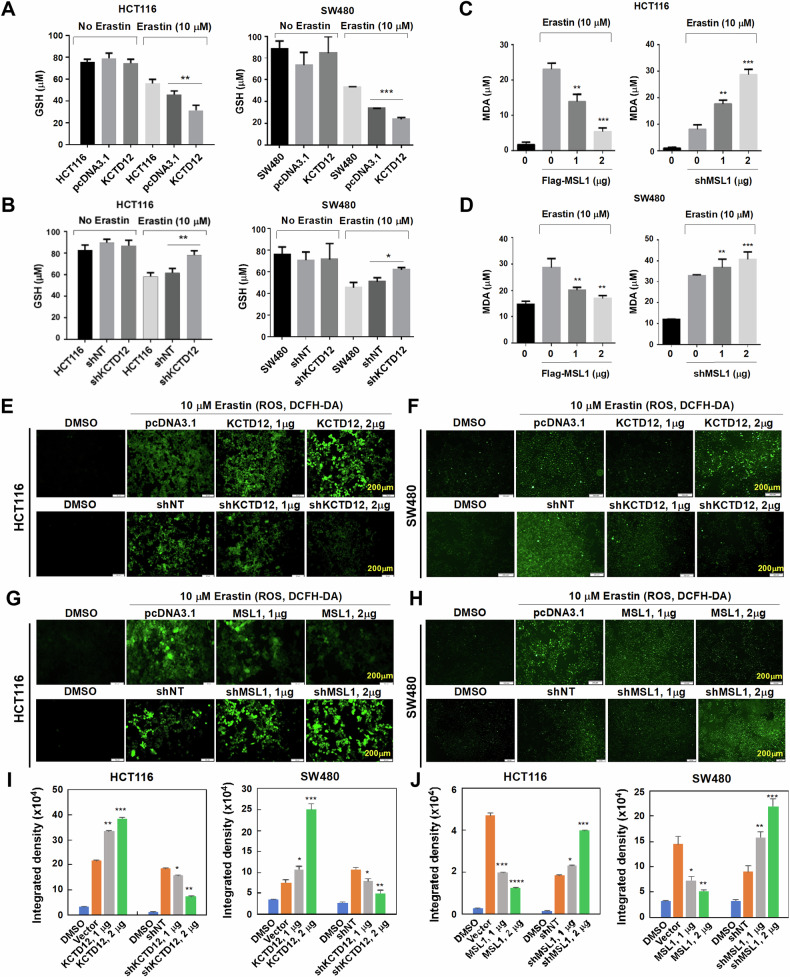
MSL1 and KCTD12 modulate GSH, ROS, and MDA levels in Erastin-treated colon cancer cells. **A**, **B** GSH levels in cells transfected with Flag-KCTD12 (0–2 µg) or pLVX-shKCTD12 (0–2 µg) under 10 µM Erastin treatment. pcDNA3.1 and pLVX-shNT served as controls. **p < 0.01 vs. pcDNA3.1 or pLVX-shNT. **C**, **D** MDA levels in cells transfected with Flag-MSL1 or pLVX-shMSL1 under 10 µM Erastin treatment. “0” indicates cells transfected with control plasmids (pcDNA3.1 or pLVX-shNT). **p < 0.01, ***p < 0.001 vs. control. **E**–**H** ROS levels in cells transfected with Flag-KCTD12, Flag-MSL1, pLVX-shKCTD12, or pLVX-shMSL1 under 10 µM Erastin treatment. DMSO was used a non-Erastin control. **I**, **J** Quantification of fluorescence intensity for (**D**) and (**F**). *p < 0.05, **p < 0.01, ***p < 0.001 vs. control.

Given the involvement of the MSL1-KCTD12-SLC7A11 axis in Erastin-induced ferroptosis, we next examined its impact on ferroptosis-associated intermediates, including ROS, GSH, and MDA. Compared to control or vector-transfected cells, KCTD12 overexpression or knockdown alone did not significantly alter GSH levels. However, following Erastin treatment, GSH levels were significantly reduced in KCTD12-overexpressing cells and increased upon KCTD12 knockdown (Fig. 6A, B). Additionally, MDA accumulation, a hallmark of lipid peroxidation, was attenuated by Flag-MSL1 overexpression (Fig. 6C, D, left panel) but was enhanced upon MSL1 knockdown with pLVX-shMSL1 in a dose-dependent manner (Fig. 6C, D, right panel).

Since lipid peroxide accumulation is a key driver of ferroptosis, we further assessed ROS levels (Fig. 6E–J). KCTD12 overexpression resulted in a dose-dependent increase in ROS levels in Erastin-treated cells, whereas KCTD12 knockdown had the opposite effect (Fig. 6E, F, upper vs. lower panels; quantified in Fig. 6I). Conversely, MSL1 overexpression suppressed ROS accumulation, while MSL1 knockdown exacerbated it in a dose dependent manner (Fig. 6G, H, quantified in Fig. 6J).

These findings further support the opposing roles of MSL1 and KCTD12 in Erastin-induced ferroptosis and reinforce the negative regulatory relationship between these two factors.

### MSL1-KCTD12 axis synergistically regulates Erastin-induced ROS, GSH, and MDA levels in colon cancer cells

**Fig. 7:**
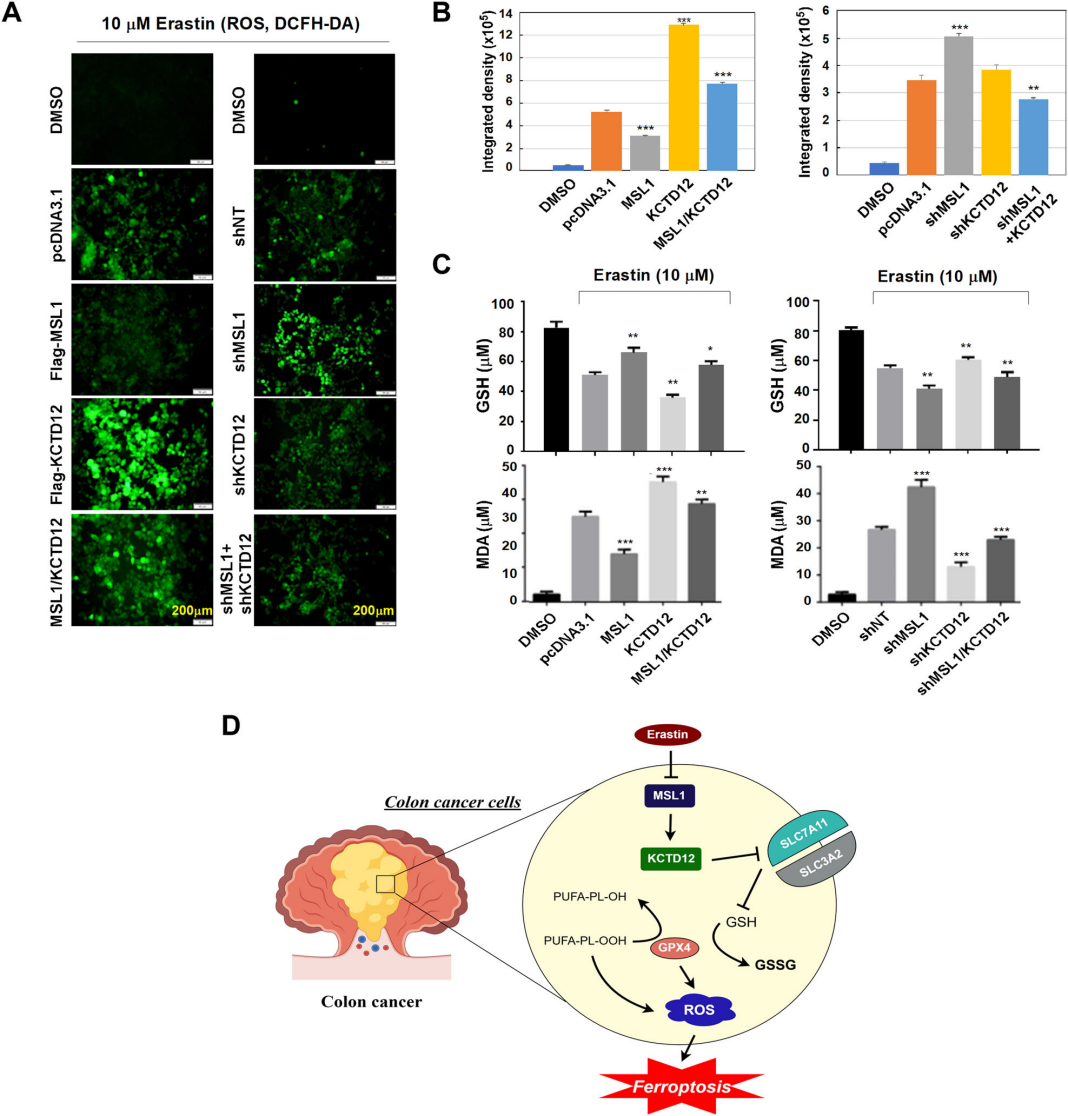
The MSL1-KCTD12 axis coordinately regulates Erastin-induced ROS, GSH, and MDA levels in HCT116 cells. **A** Intracellular ROS levels in HCT116 cells transfected with pcDNA3.1, Flag-MSL1, Flag-KCTD12, or co-transfected with Flag-MSL1 and Flag-KCTD12 under 10 µM Erastin treatment (left). Similar analysis was performed in cells with MSL1 or KCTD12 knockdown, alone or in combination (right). **B** Quantization of (**A**). **C** GSH and MDA levels in cells transfected as in (**A**) under 10 µM Erastin treatment (left). Corresponding analysis in MSL1- or KCTD12-knockdown cells (right). **D** Schematic model summarizing the findings (created with Figdraw, qPrAe09ca4). In Erastin-treated colon cancer cells, MSL1 downregulation upregulates KCTD12, leading to suppression of SLC7A11 and reduced GSH synthesis and GPX4 levels. This results in excessive intracellular lipid peroxide accumulation, increased ROS, and ferroptosis induction.

To further elucidate the interplay between MSL1 and KCTD12 in ferroptosis, we examined the effects of their simultaneous modulation on Erastin-induced ROS, GSH, and MDA levels. HCT116 cells were transfected with either Flag-MSL1 and Flag-KCTD12 alone, or co-transfected, followed by Erastin treatment. ROS levels were then assessed 24 hours post-transfection. Consistent with previous findings, Erastin-induced ROS accumulation was evident in control (pcDNA3.1 transfected) cells, and this effect was mitigated by MSL1 overexpression while exacerbated by KCTD12 overexpression. Notably, co-expression of MSL1 and KCTD12 neutralized their individual effects, restoring ROS levels to those observed in the control group (Fig. 7A, left panel). Quantified fluorescence intensity measurements are shown in Fig. 7B (left panel). Conversely, in cells transfected with pLVX-shMSL1 or pLVX-shKCTD12 alone or in combination, the opposite effects were observed (Fig. 7A, right panel; quantified in Fig. 7B, right panel).

Next, we assessed GSH and MDA levels under the same conditions. As shown in Fig. 7C (upper panel), Erastin-induced GSH production was enhanced by MSL1 overexpression and suppressed by MSL1 knockdown. In contrast, KCTD12 overexpression reduced GSH levels, whereas its knockdown promoted GSH accumulation. When both MSL1 and KCTD12 were simultaneously overexpressed or knocked down, their opposing effects canceled each other out, restoring GSH levels to baseline. Similarly, MDA accumulation in Erastin-treated cells (relative to the DMSO control) was negatively regulated by MSL1 and positively regulated by KCTD12 (Fig. 7C, lower panel). Again, co-modulation of both proteins neutralized their individual effects on MDA content. These findings highlight the opposing regulatory roles of MSL1 and KCTD12 in ferroptosis and suggest that their interplay dictates the balance of ROS, GSH, and MDA levels in response to Erastin treatment.

## Discussion

In this study, we first confirmed the negative regulatory effect of MSL1 on KCTD12. Furthermore, we found that MSL1 downregulation, via modulation of KCTD12, suppresses the expression of key negative regulators of ferroptosis, SLC7A11 and GPX4, thereby promoting Erastin-induced ferroptosis in colon cancer cells. These findings suggest that the MSL1-KCTD12-SLC7A11 axis represents a potential regulatory mechanism underlying ferroptosis in colon cancer (Fig. 7D).

MSL1 is a crucial structural composition of the MOF/MSL complex, maintaining its allosteric activity [4]. Moreover, MSL1 is highly expressed in various cancer tissues and has been implicated in tumor cell survival through multiple mechanisms [31]. To date, only one study has examined MSL1’s role in colon cancer, demonstrating that MSL1 knockdown enhances cisplatin-induced DNA damage, promote apoptosis in HCT116 cells, and thereby facilitates tumor cell elimination [6]. Consistent with this, we observed that MSL1 depletion was strongly associated with disrupted cell division.

Given MSL1’s oncogenic role, we further investigated its downstream regulatory mechanisms and identified KCTD12 as a potential target of the MSL1/MSL3. Abnormal KCTD12 expression is closely linked to tumor development [11, 12], and we confirmed its downregulation in primary colon cancer tissues. Notably, reduced KCTD12 expression enhances the stemness of colorectal cancer cells—promoting self-renewal, tumorigenesis, and drug resistance—through modulation of the ERK pathway downstream of GABA signaling, leading to poor prognosis [32]. Our study is the first to establish a negative regulatory relationship between MSL1 and KCTD12.

Both MSL1 and KCTD12 have been implicated in mitochondrial function. MSL1 can be activated by increased mitochondrial membrane potential, triggering ROS production and lipid peroxidation [33], while KCTD12 is considered a key gene associated with hypoxia and mitochondrial scoring [34]. These findings provide indirect evidence for their potential interaction.

Recent studies have increasingly focused on the role of ferroptosis in colon cancer [17, 35, 36]. Ferroptosis, a form of programmed cell death driven by the excessive accumulation of iron-dependent lipid peroxides, has emerged as a promising therapeutic strategy for colon cancer [37]. Multiple factors modulate ferroptosis during colon carcinogenesis [18, 38], yet MSL1’s role in this process remains unexplored. In this study, we found that Erastin-induced ferroptosis in colon cancer cells significantly reduced MSL1 expression, suppressing the H4K16ac enzyme activity of the MOF/MSL complex and upregulating KCTD12 expression. Further, we demonstrated that MSL1 functions as a negative regulator of ferroptosis, whereas KCTD12 acts as a positive regulator. Importantly, the inhibitory effect of MSL1 on ferroptosis was partially reverted by KCTD12, highlighting the regulatory role of the MSL1-KCTD12 axis ferroptosis. Consistent with this hypothesis, transcriptome analysis of MSL1-knockout cells identified 34 differentially expressed genes in the SLC family, including SLC38A1, SLC16A1, SLC7A11, and SLC3A2, all of which are closely associated with ferroptosis [39]. Furthermore, we revealed that under Erastin treatment, the MSL1-KCTD12 axis promotes ferroptosis, at least in part, by regulating SLC7A11 expression.

SLC7A11, a key inhibitor of ferroptosis, mediates cystine uptake and promotes glutathione synthesis [40]. Erastin binds to the xCT-4F2hc complex, where its chlorophenoxy group interacts with the Phe254 residue in xCT TM6b, inhibiting xCT activity, depleting intracellular cysteine and glutathione, and inducing ferroptosis [41]. While direct interactions between KCTD12 and SLC7A11 remain poorly characterized. Zhou et al. reported that cullin-3 ligase inactivation increases cysteine uptake and SLC7A11 accumulation [42], suggesting that KCTD12 may negatively regulate SLC7A11.

However, the specific molecular mechanism by which Erastin downregulates MSL1 remains unclear. Beyond inhibiting the Xc-system, Erastin also targets p53 in tumor cells [43]. Erastin-induced ROS can activate p53 and its downstream pathways, further amplifying oxidative stress and ferroptosis. Notably, Erastin does not affect p53 in normal lung cells, indicating its tumor cell-specific regulation [43]. Previous studies have shown that the MOF-MSL1v1 complex can specifically bind p53, and MSL1 preferentially associates with transcription start sites [44]. Additionally, our molecular docking analysis identified binding sites between MSL1 and Erastin, suggesting that Erastin may directly inhibit MSL1 and promote ferroptosis. Based on these findings, we propose two potential mechanisms for Erastin-induced MSL1 downregulation: (1) indirect transcriptional suppression via p53 or (2) direct targeting of MSL1 as a molecular substrate.

To further validate our findings, we employed an alternative ferroptosis model using the small-molecule inhibitor Fer-1, which mitigates ferroptosis by scavenging alkoxyl radicals and lipid peroxidation products [45]. However, the impact of Fer-1 on the Erastin-induced MSL1/KCTD12/SLC7A11/GPX4 axis remains largely unknown. Future studies will aim to elucidate the underlying mechanisms, providing new therapeutic targets and a theoretical basis for clinical interventions in colon cancer.

## Supplementary information

Original Data

## Data Availability

All data used in this work can be acquired from the TCGA database (http://cancergenome.nih.gov/), ENCODE database (http://encodeproject.org/), CCLE database (http://sites.broadinstitute.org/ccle), Gene Expression Omnibus (GEO) datasets (https://www.ncbi.nlm.nih.gov/geo/), GTEx project (https://gtexportal.org/home/). Raw and processed RNA-seq data of H520 cells transfected with Gapmer ASOs or deleted for *NFYC-AS1* TSS have been deposited at GEO under accession number GSE240468 and are available from the corresponding author upon reasonable request.
