# Supplementary material for: Male-specific lethal 1 (MSL1) promotes Erastin-induced ferroptosis in colon cancer cells by regulating the KCTD12-SLC7A11 axis
Source: Cell Death Dis. 2025 Apr 12;16(1):281. doi: 10.1038/s41419-025-07555-7 (PMC11993775; doi:10.1038/s41419-025-07555-7)
Supplement: Supplementary file 1 — Original Data [file 41419_2025_7555_MOESM1_ESM.pptx]

## Slide 1
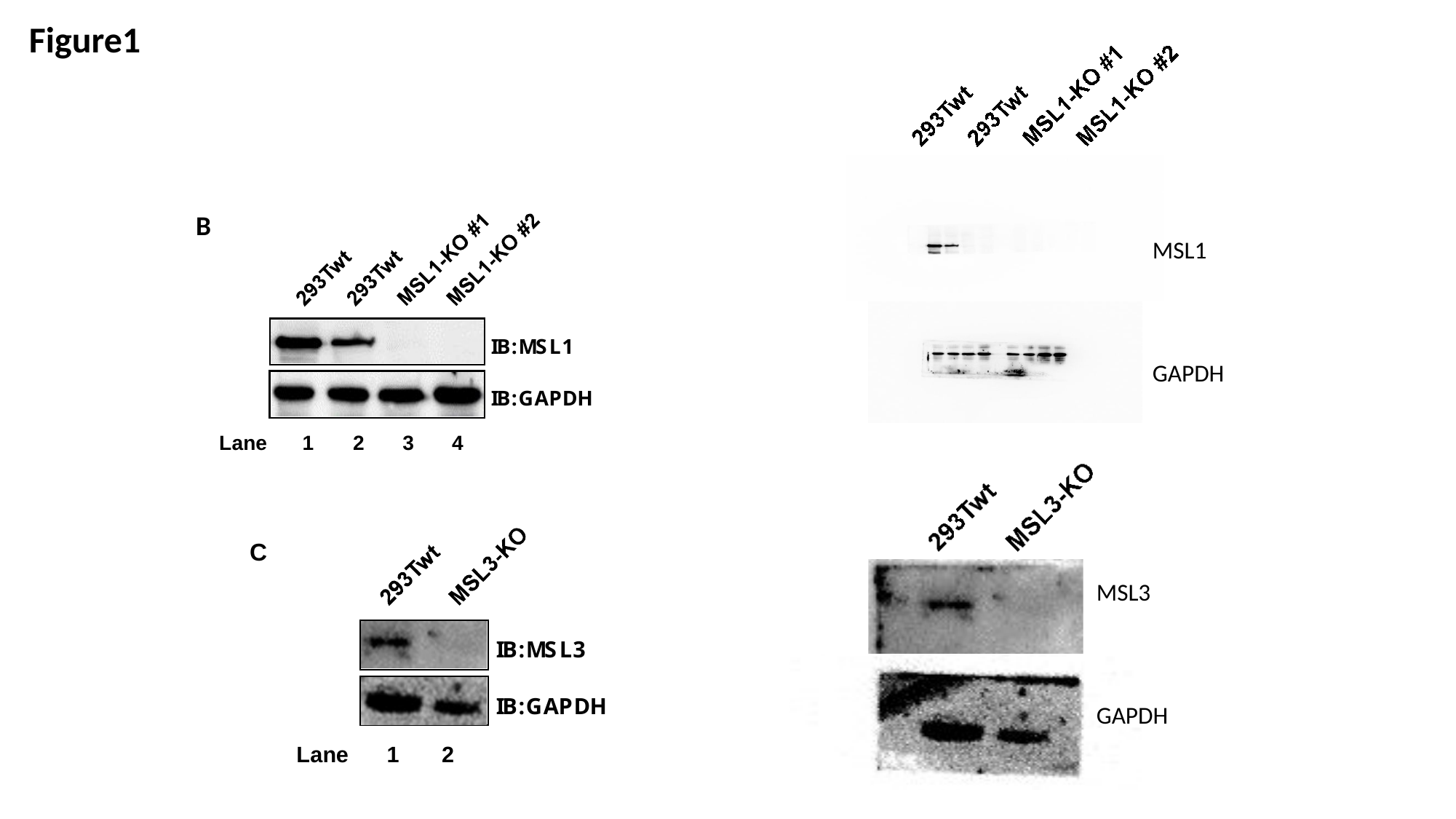

Figure1
B
MSL1
GAPDH
C
MSL3
GAPDH

## Slide 2
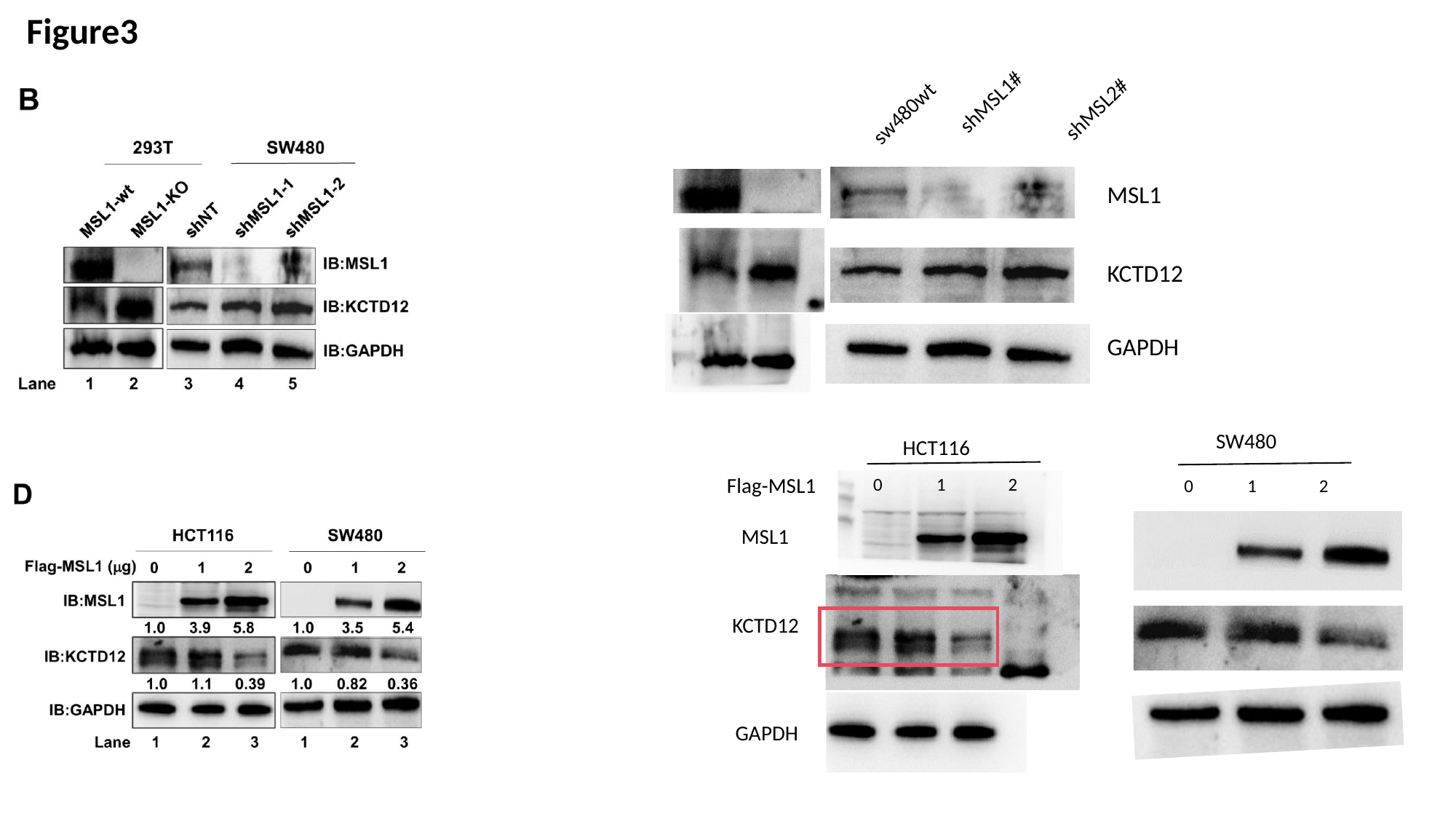

Figure3
shMSL1#
shMSL2#
sw480wt
MSL1
KCTD12
GAPDH
SW480
0 1 2
HCT116
Flag-MSL1
0 1 2
MSL1
KCTD12
GAPDH

## Slide 3
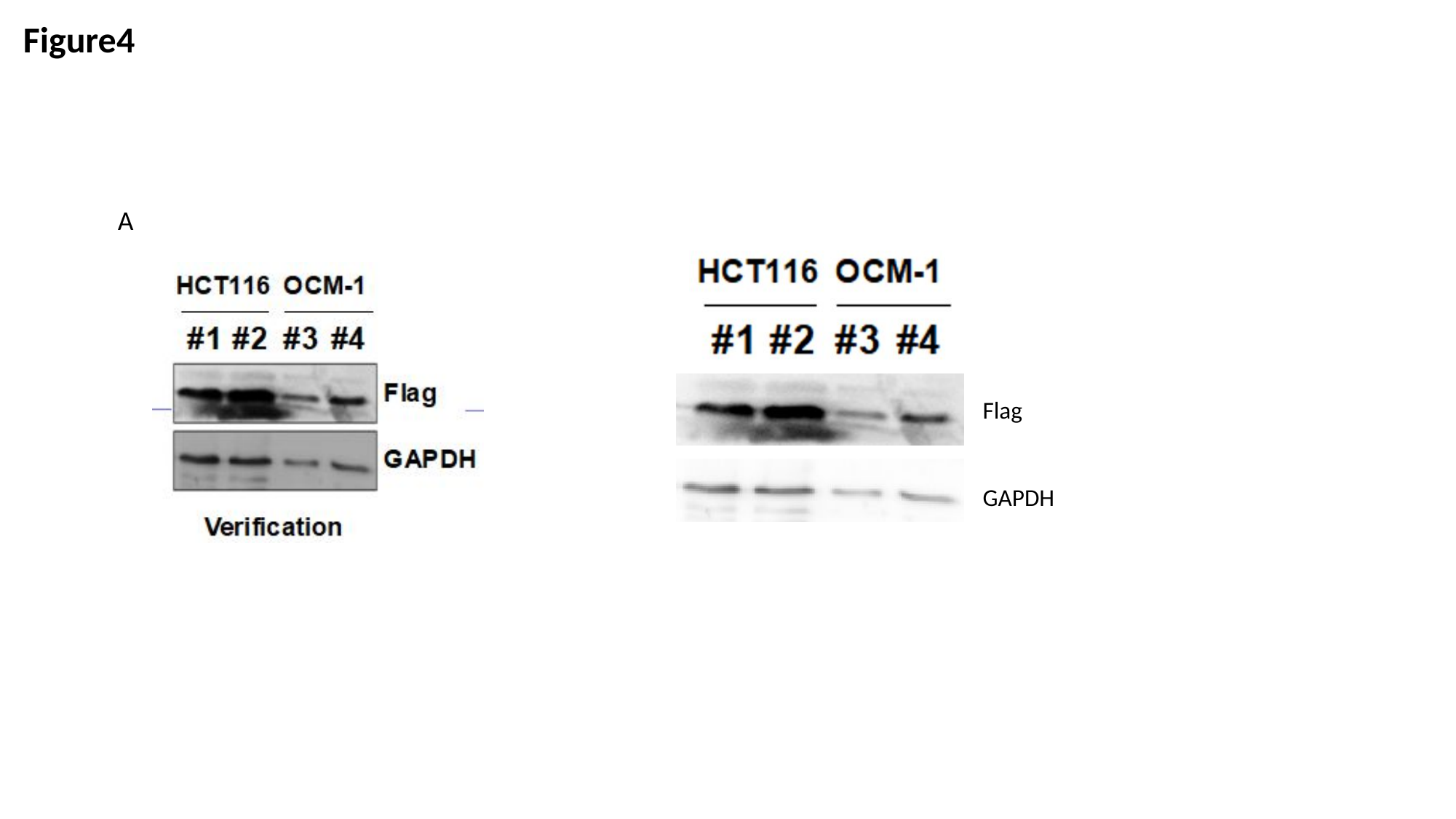

Figure4
A
Flag
GAPDH

## Slide 4
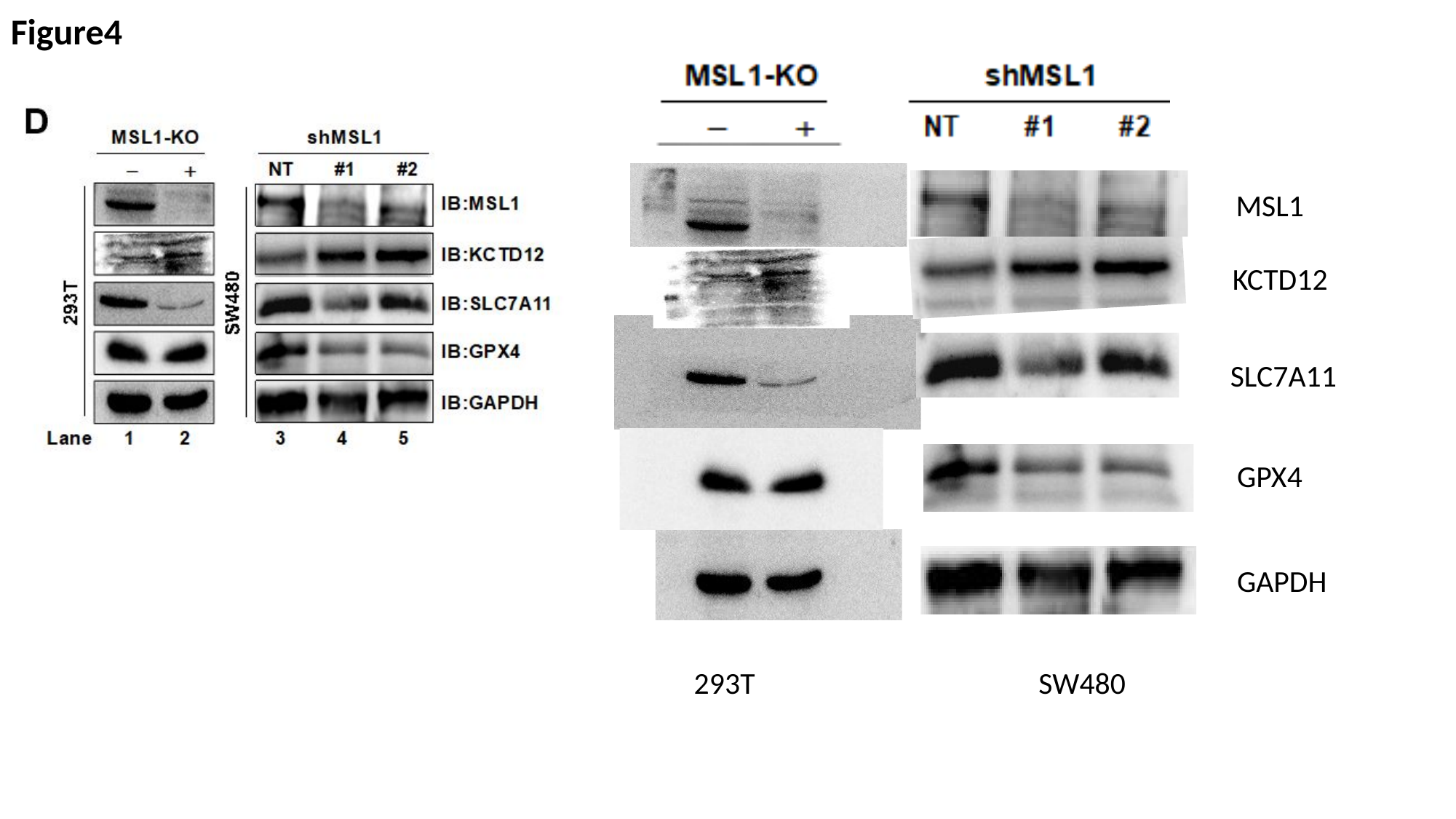

Figure4
MSL1
KCTD12
SLC7A11
GPX4
GAPDH
293T SW480

## Slide 5
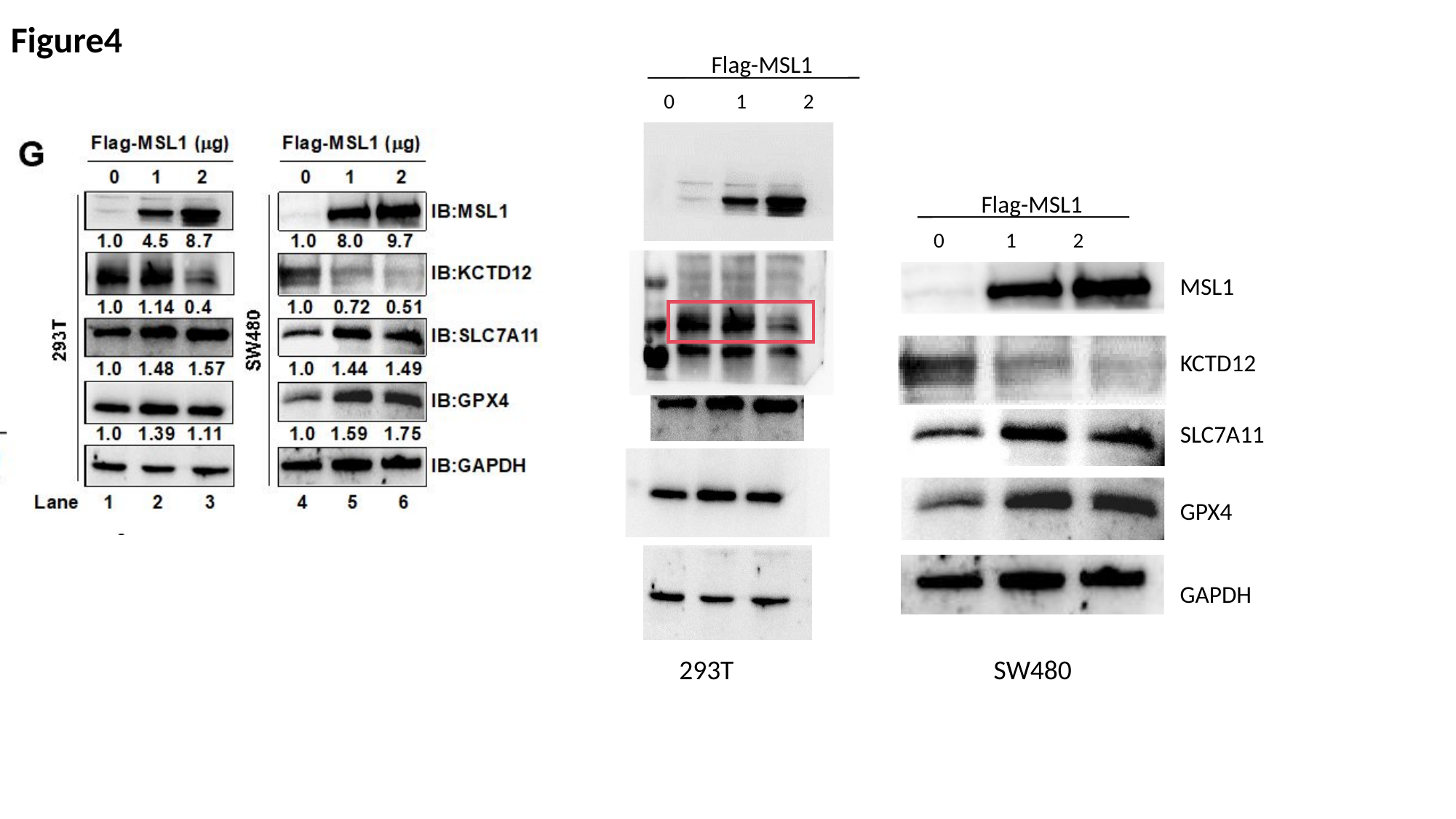

Figure4
Flag-MSL1
0 1 2
Flag-MSL1
0 1 2
MSL1
KCTD12
SLC7A11
GPX4
GAPDH
293T SW480

## Slide 6
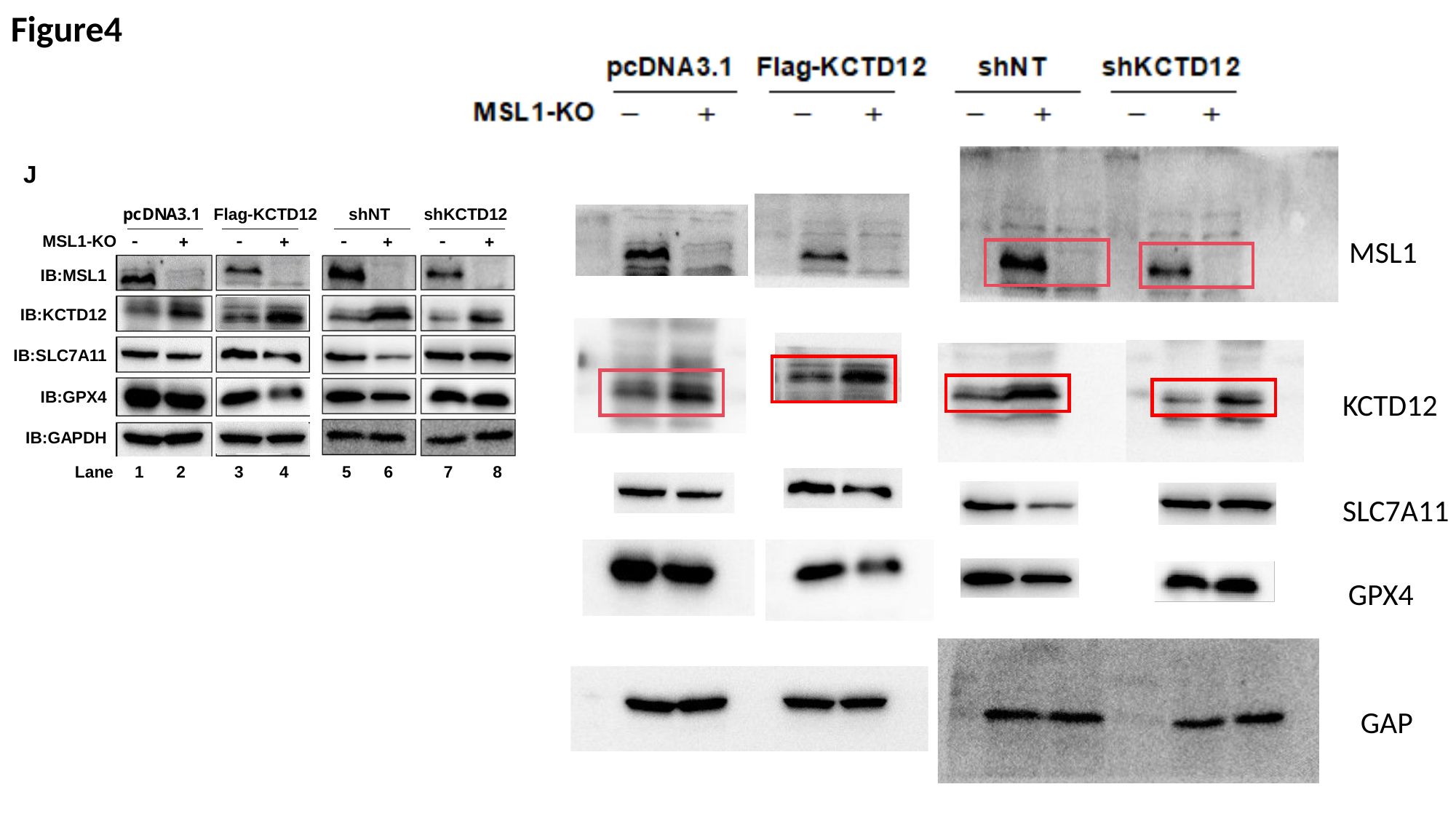

Figure4
MSL1
KCTD12
SLC7A11
GPX4
GAP
J

## Slide 7
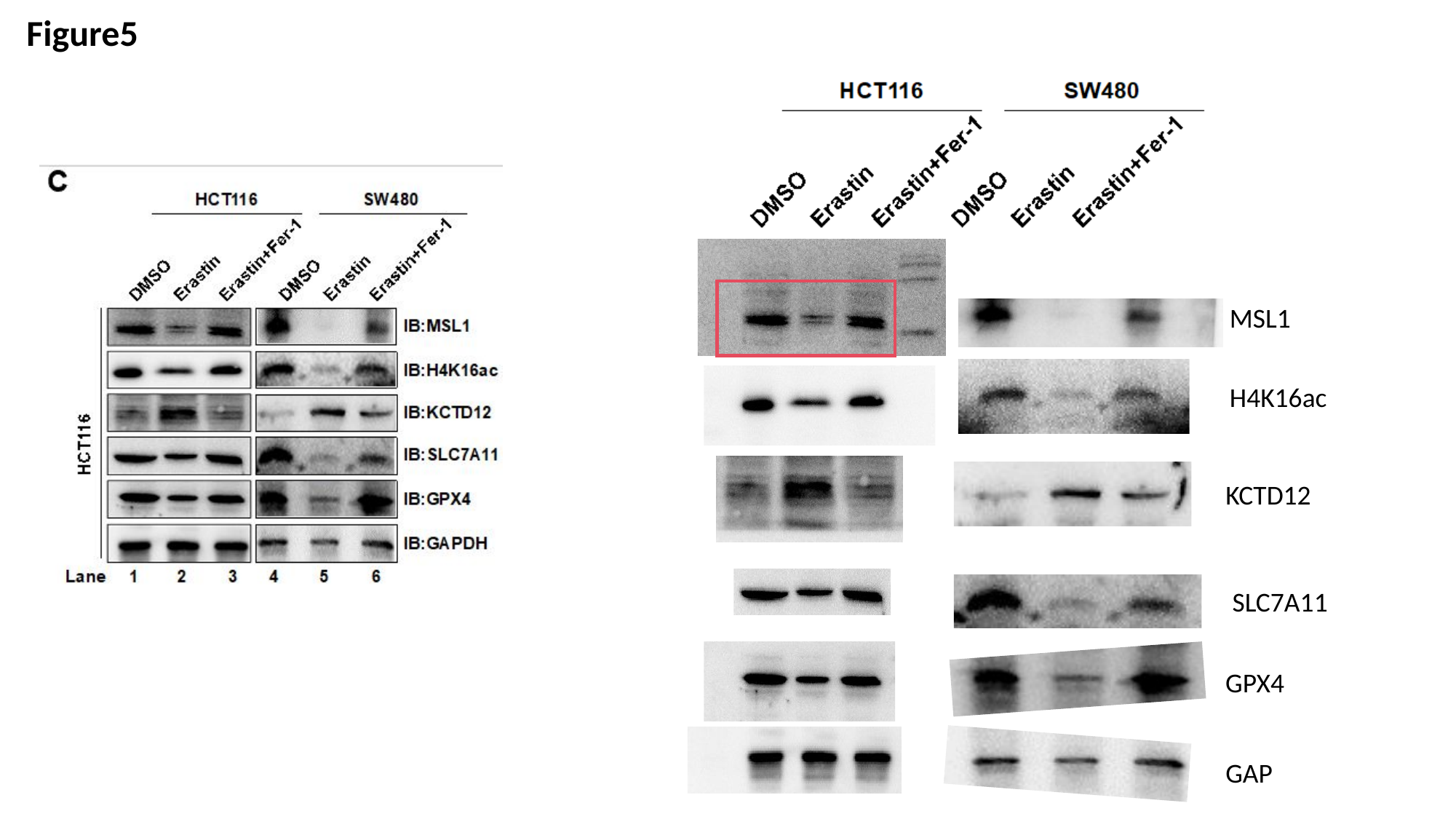

Figure5
MSL1
H4K16ac
KCTD12
SLC7A11
GPX4
GAP

## Slide 8
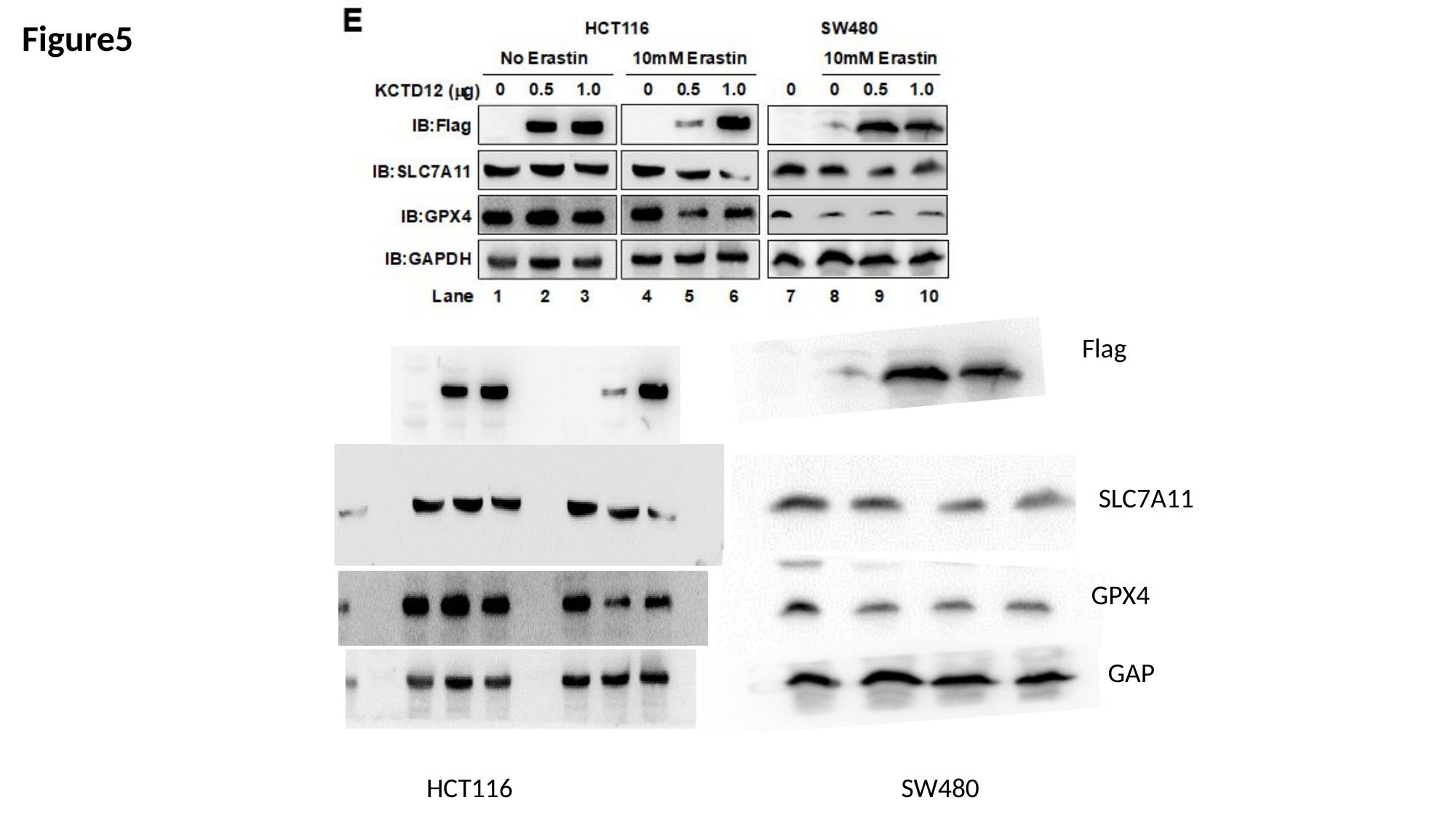

Figure5
Flag
SLC7A11
GPX4
GAP
HCT116 SW480

## Slide 9
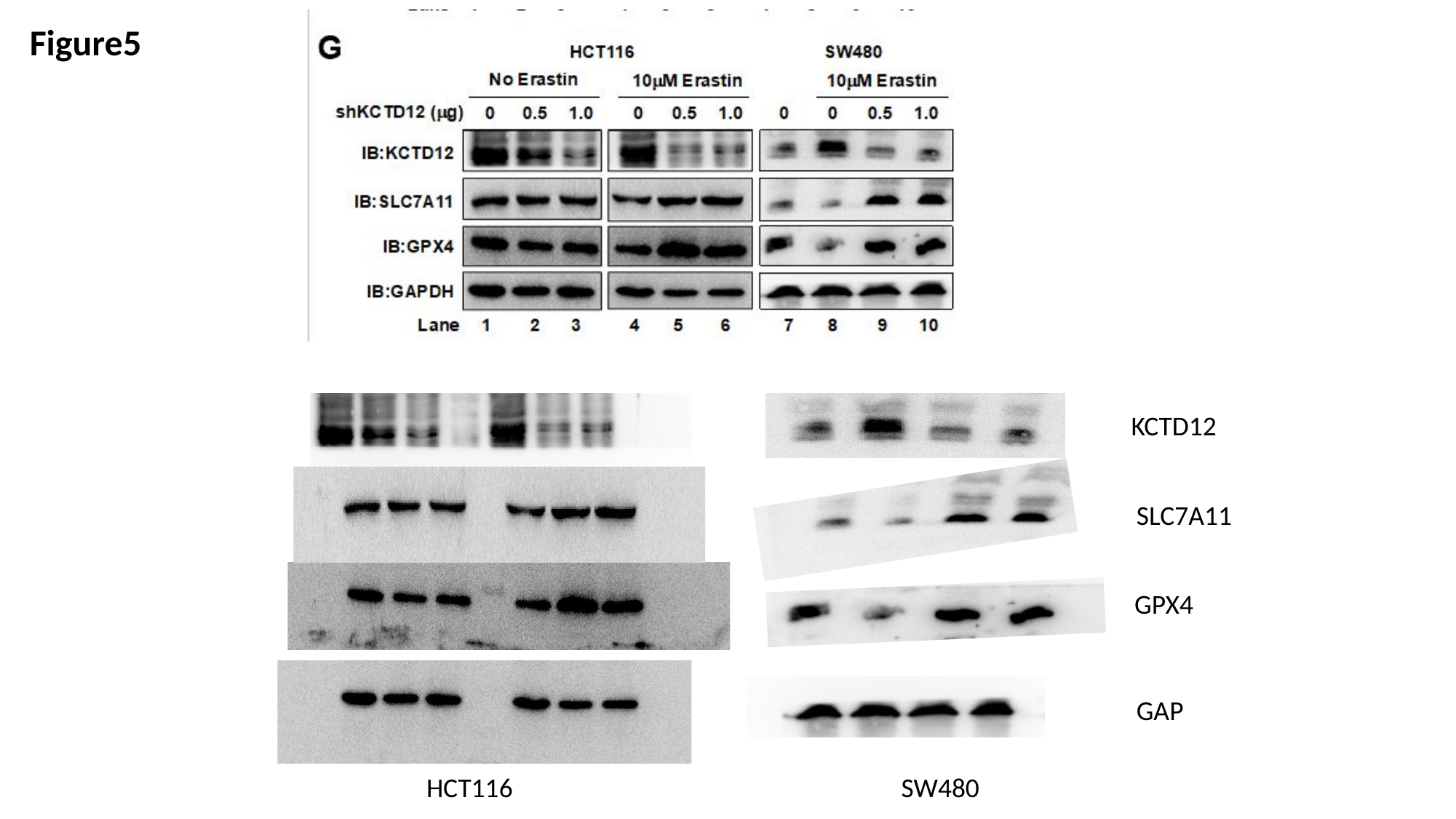

Figure5
KCTD12
SLC7A11
GPX4
GAP
HCT116 SW480

## Slide 10
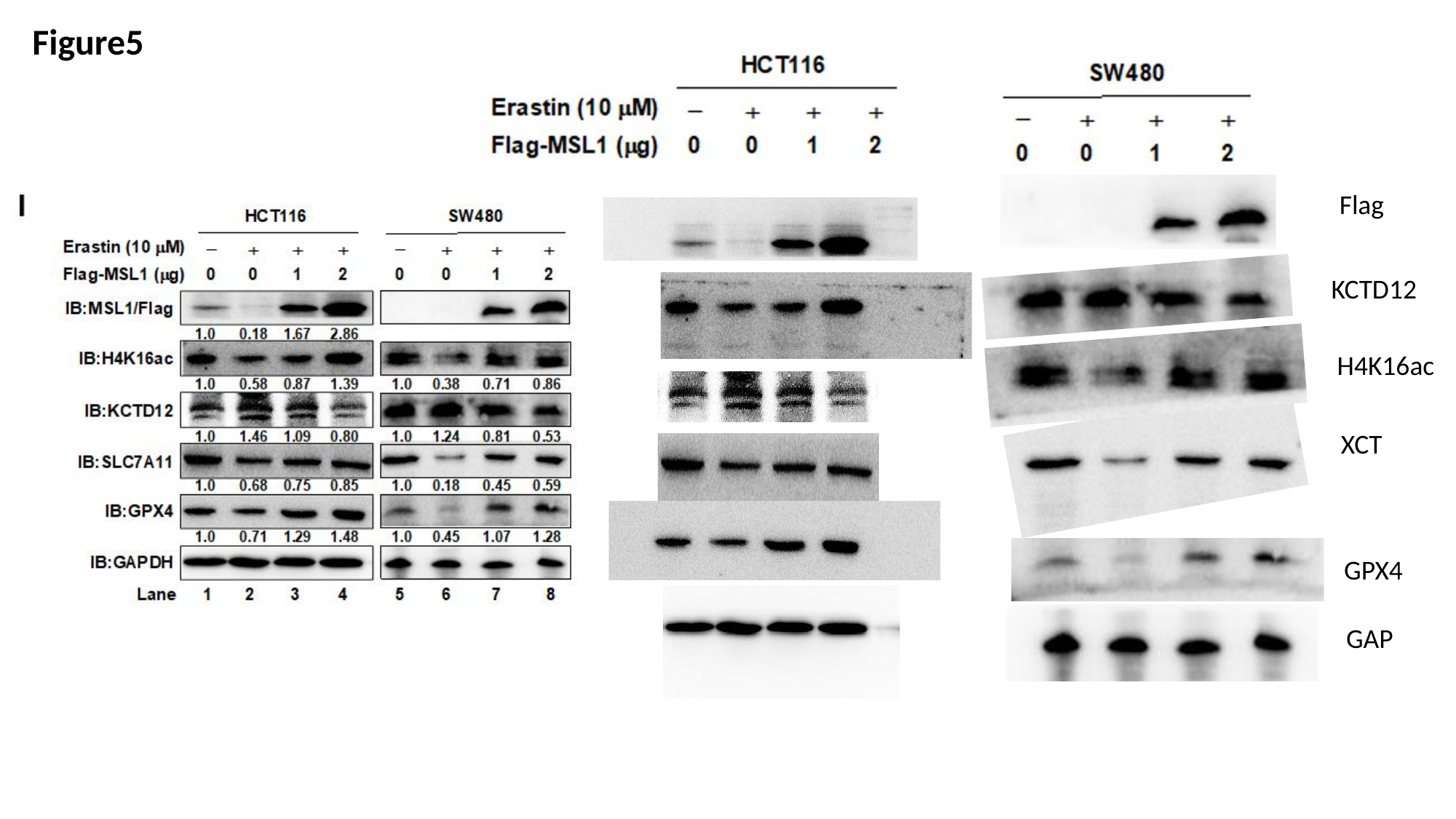

Figure5
Flag
KCTD12
H4K16ac
XCT
GPX4
GAP

## Slide 11
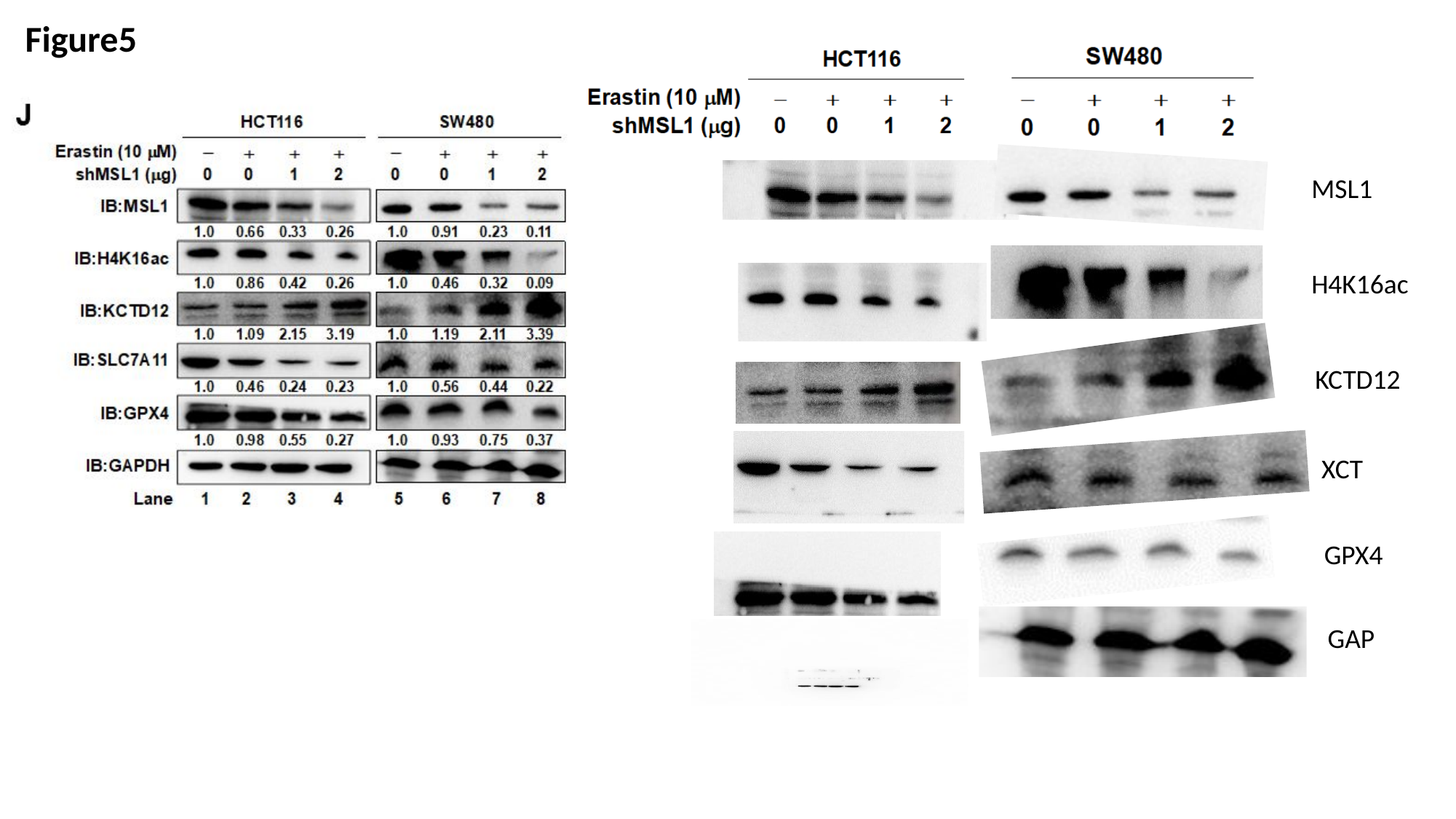

Figure5
MSL1
H4K16ac
KCTD12
XCT
GPX4
GAP
